# Supplementary material for: Post-Exercise Protein Intake May Reduce Time in Hypoglycemia Following Moderate-Intensity Continuous Exercise among Adults with Type 1 Diabetes
Source: Nutrients. 2023 Oct 6;15(19):4268. doi: 10.3390/nu15194268 (PMC10574378; doi:10.3390/nu15194268)
Supplement: Supplementary file 1 [file nutrients-15-04268-s001.zip › Supplementary Figure Captions.pdf]

Supplementary Figure Captions:

Supplementary Figure S1. Scatter Plot of Individual Responses to Post-Exercise Protein Intake (g/kg) on Post-Exercise Percent Time Above Range (>180mg/dL) Following Moderate-Intensity Continuous Training (MICT).

Supplementary Figure S2. Scatter Plot of Individual Responses to Post-Exercise Protein Intake (g/kg) on Post-Exercise Percent Time Above Range (>180mg/dL) Following High-Intensity Interval Training (HIIT).

Supplementary Figure S3. Scatter Plot of Individual Responses to Post-Exercise Protein Intake (grams) on Post-Exercise Percent Time Above Range (>180mg/dL) Following Moderate-Intensity Continuous Training (MICT).

Supplementary Figure S4. Scatter Plot of Individual Responses to Post-Exercise Protein Intake (grams) on Post-Exercise Percent Time Above Range (>180mg/dL) Following High-Intensity Interval Training (HIIT).

Supplementary Figure S5. Scatter Plot of Individual Responses to Post-Exercise Protein Intake (g/kg) on Post-Exercise Percent Time In Range (70 - 180mg/dL) Following Moderate-Intensity Continuous Training (MICT).

Supplementary Figure S6. Scatter Plot of Individual Responses to Post-Exercise Protein Intake (g/kg) on Post-Exercise Percent Time In Range (70 - 180mg/dL) Following High-Intensity Interval Training (HIIT).

Supplementary Figure S7. Scatter Plot of Individual Responses to Post-Exercise Protein Intake (grams) on Post-Exercise Percent Time In Range (70 - 180mg/dL) Following Moderate-Intensity Continuous Training (MICT).

Supplementary Figure S8. Scatter Plot of Individual Responses to Post-Exercise Protein Intake (grams) on Post-Exercise Percent Time In Range (70 - 180mg/dL) Following High-Intensity Interval Training (HIIT).

Supplementary Figure S9. Scatter Plot of Individual Responses to Post-Exercise Protein Intake (g/kg) on Post-Exercise Percent Time Below Range (<70 mg/dL) Following Moderate-Intensity Continuous Training (MICT).

Supplementary Figure S10. Scatter Plot of Individual Responses to Post-Exercise Protein Intake (g/kg) on Post-Exercise Percent Time Below Range (<70 mg/dL) Following High-Intensity Interval Training (HIIT).

Supplementary Figure S11. Scatter Plot of Individual Responses to Post-Exercise Protein Intake (grams) on Post-Exercise Percent Time Below Range (<70 mg/dL) Following Moderate-Intensity Continuous Training (MICT).

Supplementary Figure S12. Scatter Plot of Individual Responses to Post-Exercise Protein Intake (grams) on Post-Exercise Percent Time Below Range (<70 mg/dL) Following High Intensity Interval Training (HIIT).
