# Supplementary figures and images for: Post-Exercise Protein Intake May Reduce Time in Hypoglycemia Following Moderate-Intensity Continuous Exercise among Adults with Type 1 Diabetes
Source: Nutrients. 2023 Oct 6;15(19):4268. doi: 10.3390/nu15194268 (PMC10574378; doi:10.3390/nu15194268)

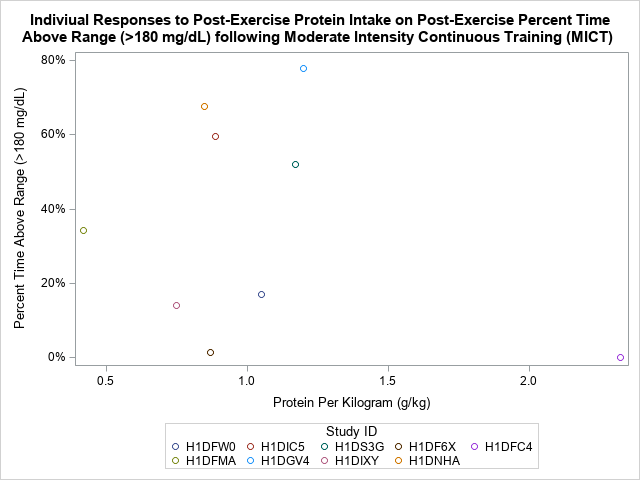

Supplement: Supplementary file 1 [file nutrients-15-04268-s001.zip › Supplementary Figure S1.png]

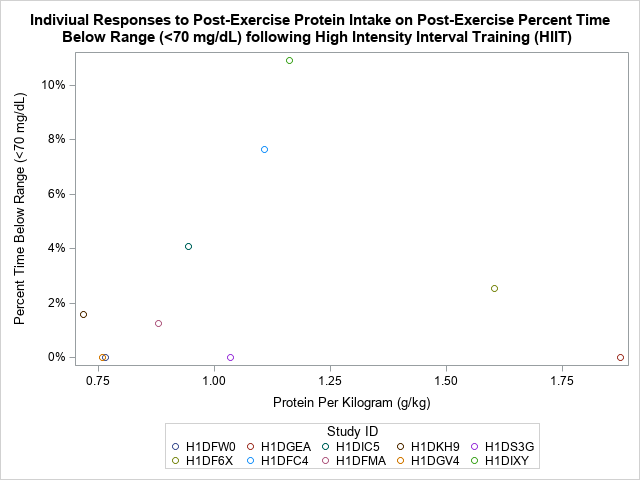

Supplement: Supplementary file 1 [file nutrients-15-04268-s001.zip › Supplementary Figure S10.png]

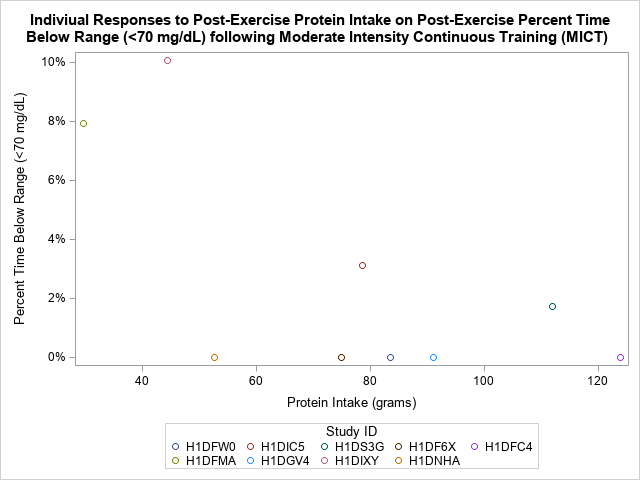

Supplement: Supplementary file 1 [file nutrients-15-04268-s001.zip › Supplementary Figure S11.png]

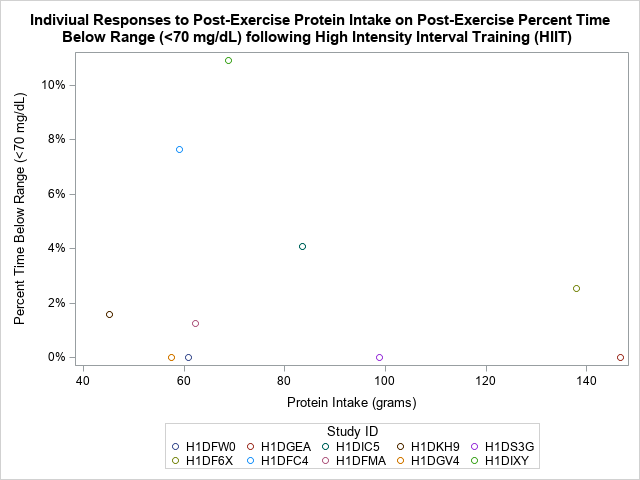

Supplement: Supplementary file 1 [file nutrients-15-04268-s001.zip › Supplementary Figure S12.png]

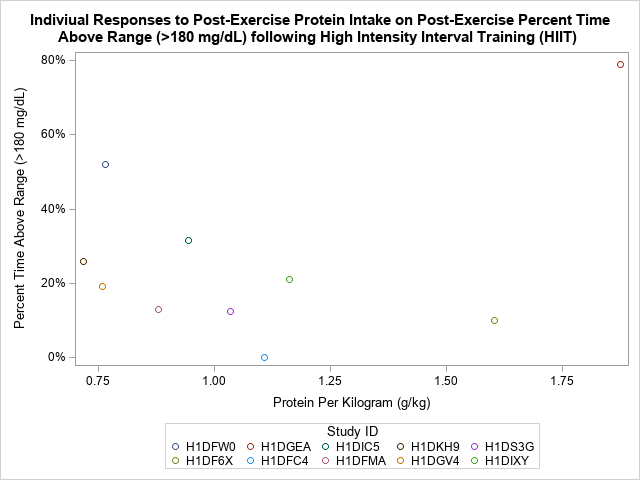

Supplement: Supplementary file 1 [file nutrients-15-04268-s001.zip › Supplementary Figure S2.png]

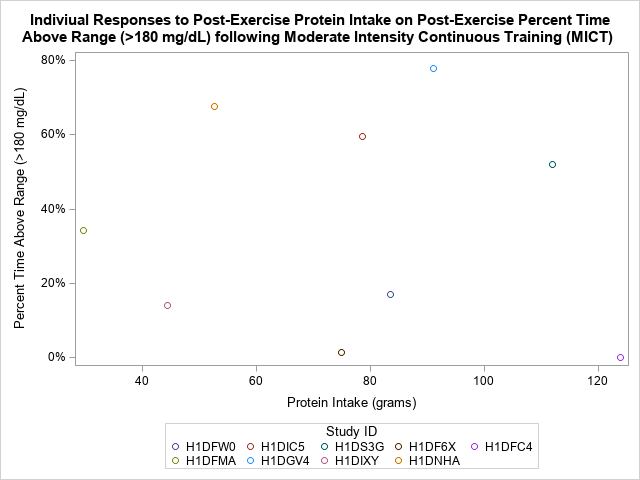

Supplement: Supplementary file 1 [file nutrients-15-04268-s001.zip › Supplementary Figure S3.png]

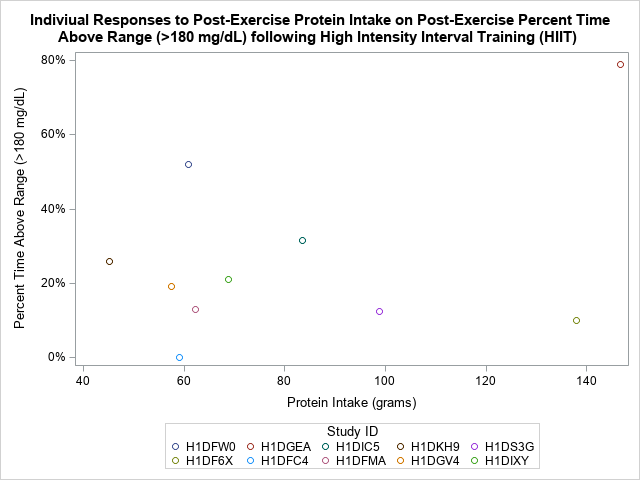

Supplement: Supplementary file 1 [file nutrients-15-04268-s001.zip › Supplementary Figure S4.png]

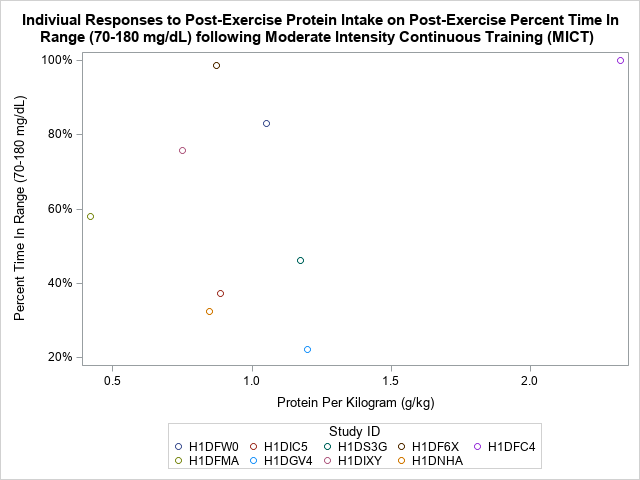

Supplement: Supplementary file 1 [file nutrients-15-04268-s001.zip › Supplementary Figure S5.png]

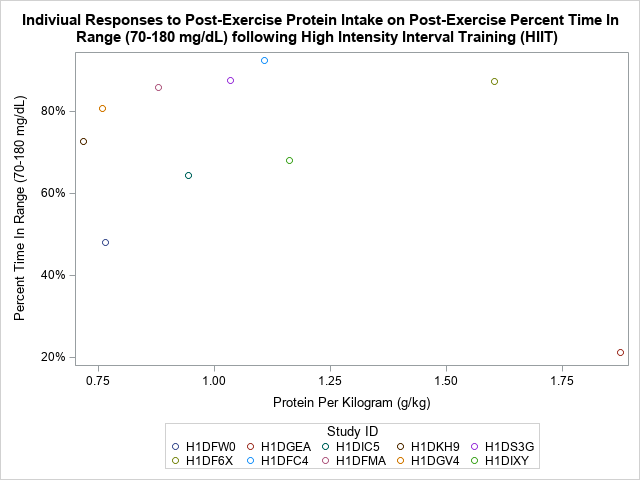

Supplement: Supplementary file 1 [file nutrients-15-04268-s001.zip › Supplementary Figure S6.png]

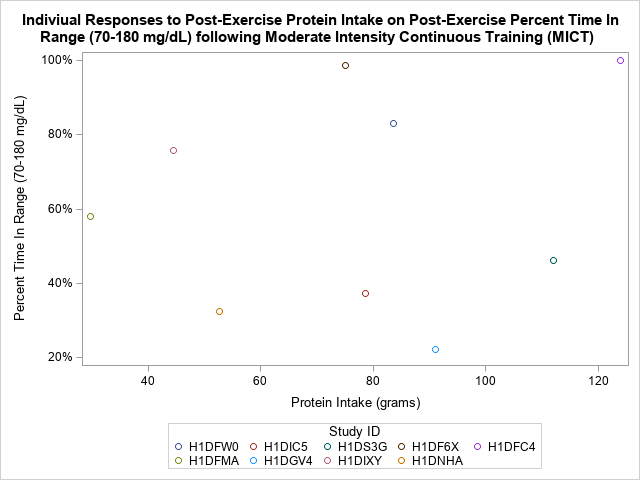

Supplement: Supplementary file 1 [file nutrients-15-04268-s001.zip › Supplementary Figure S7.png]

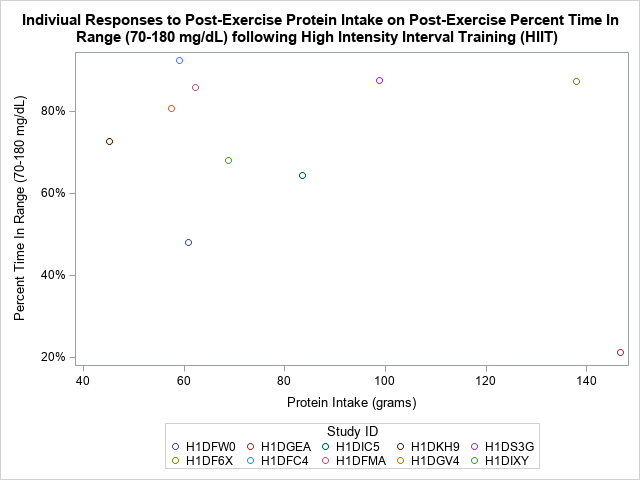

Supplement: Supplementary file 1 [file nutrients-15-04268-s001.zip › Supplementary Figure S8.png]

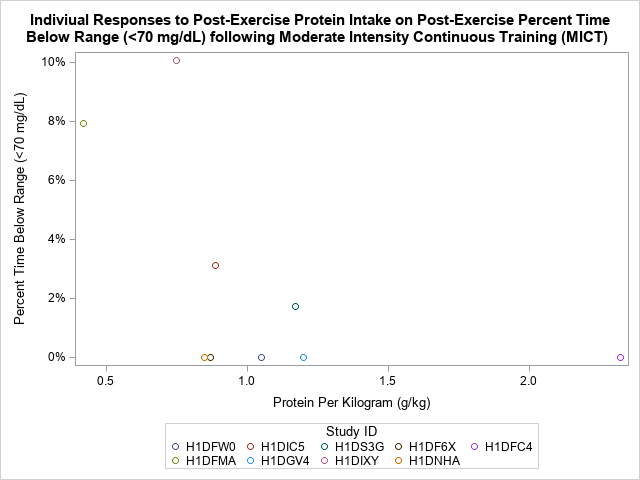

Supplement: Supplementary file 1 [file nutrients-15-04268-s001.zip › Supplementary Figure S9.png]
